# How well did the consensus methods apply in the guideline development of traditional Chinese medicine: a web-based survey in China

**DOI:** 10.1186/s12874-023-02087-0

**Published:** 2023-11-10

**Authors:** Changhao Liang, Guanxiang Yin, Ziyi Lin, Jing Cui, Yaqi Wang, Siqi Liu, Dingran Yin, Pengwei Liu, Xiangfei Su, Hongguo Rong, Cheng Wang, Feng Sun, Yutong Fei

**Affiliations:** 1 Centre for Evidence-Based Chinese Medicine, Beijing University of Chinese Medicine, No. 11, North Third Ring Road, Chaoyang District, Beijing, 100029 China; 2 Beijing University of Chinese Medicine, Beijing, 100029 China; 3 Chinese Society of Traditional Chinese Medicine, Beijing, 100029 China; 4 Xinjiang Uygur Autonomous Region Academy of Traditional Chinese Medicine, Xinjiang, 830001 China; 5 Department of Epidemiology and Biostatistics, School of Public Health, Peking University, Beijing, 100191 China

**Keywords:** Research Methodology, Consensus Method, Clinical practice guidelines, Evidence-based Medicine, Expert Opinion, Surveys and questionnaires

## Abstract

**Background and objective:**

Consensus methods are crucial in developing clinical guidelines. Different methods, such as the Delphi and nominal group techniques, are commonly used, but there is a lack of detailed instructions on how to implement them effectively. The survey aims to explore the opinions and attitudes of the chair, panel and working group on the critical elements of the consensus methods during guideline development.

**Methods:**

We used a cross-sectional design to conduct this study and sent a structured questionnaire to stakeholders, including the chair, panel members, and working group participants, through the popular mobile phone application WeChat.We selected participants using a combination of purposive and snowball sampling. The questionnaire gathered information on demographics, experiences, opinions, and concerns regarding consensus methods and guideline development.

**Results:**

The sample comprised 290 participants representing 31 provinces or municipalities. Among them, the most significant number of respondents (n = 107, 36.9%) were from Beijing. Most participants, specifically 211 (72.76%), held senior professional titles, while 186 (64.14%) adhered to ongoing guidelines. The Delphi method was the most commonly used consensus method (n = 132, 42.31%), but the respondents had only a preliminary understanding of it (n = 147, 47.12%). The consensus process also revealed the insufficiency of involving pharmacoeconomists, patients, and nurses.

**Conclusions:**

Consensus methods have to be standardised and used consistently in the guideline development process. The findings of this study offer insights into diverse roles and more effective ways to apply the consensus process during guideline development.

**Supplementary Information:**

The online version contains supplementary material available at 10.1186/s12874-023-02087-0.

## Introduction

The normative application of consensus methods is fundamental in ensuring the scientific validity and reliability of recommendations within evidence-based clinical practice guidelines. Consensus methods refer to systematic approaches for summarising expert opinions, establishing agreements, and constructing recommendations [1]. Theoretically, collective decision-making offers a wide range of benefits, such as accessing a broader range of knowledge and experience, considering diverse options that arise from interactions among members, and exerting a more substantial influence on the behaviour of individual members as a cohesive group [2, 3]. Regardless of whether sufficient research evidence exists, utilising a consensus approach is essential in integrating the viewpoints of all relevant stakeholders to facilitate informed decision-making [4]. The Delphi method, modified Delphi method, nominal group technique (NGT), and consensus development conference are formal consensus methods usually used in guidelines [5]. During the development of guidelines, two key stages to which the consensus methods are emphasized: defining clinical questions and outcome indicators, as well as making recommendations. To reach a consensus during these stages, the Delphi method or modified Delphi method is frequently utilized, and face-to-face discussions may also take place. Guideline chairs guide the process, promote discussion, and oversee development. Panel members bring expertise, provide feedback, and work collaboratively.Working group participants review evidence, compile necessary materials, and arrange meetings. After the guideline draft is finished, reviewers engage in peer review of the content and development process. Although previous literature has discussed the similarities and differences as well as the basic process of consensus methods [6–12], there remains a lack of detailed instructions on how to design and implement them effectively.

Consensus methodology is particularly relevant in addressing the unique challenges posed by the diverse and multifaceted nature of traditional Chinese medicine (TCM), given the limited number of high-quality randomized controlled trials (RCTs) in this field. This underscores the imperative for a cautious and standardized application of consensus methodology in the development of clinical practice guidelines for TCM.

TCM is an essential part of China’s healthcare system and has been officially identified and integrated into the national health insurance system. Various associations have developed clinical practice guidelines for TCM, and 2017–2021 published 305 guidelines [13]. The China Association of Chinese Medicine (CACM) is the leading academic association for developing TCM guidelines in China, producing 61.05% of guidelines or consensus statements in 2016–2020 [14].

This study investigated the current consensus process in developing clinical practice guidelines in TCM via online Chinese surveys. Specifically, we examined participants’ understanding of consensus methods, their viewpoints on factors that could affect the reliability of consensus results, and strategies to achieve a more scientific and objective consensus.

## Methods

### Participants and recruitment

We recruited participants via the WeChat mobile application using purposive and snowball sampling methods. Individuals eligible for participation had experience in leading the development of clinical practice guidelines (CPGs), participating in the consensus process within a guideline working group or panel, or serving as a reviewer of guidelines. We initially sent invitations via WeChat to five CACM guideline development training groups with 842 members. The sample size calculations were based on a margin of error of 5% and assumed that the proportion of individuals with consensus experience was 80%. These estimates show a target sample size of 246 practitioners from the CACM training groups. We extended invitations to chairs and members of guideline working groups who were involved in developing or had already completed guideline projects under the management of CACM between 2020 and 2021. Moreover, group members were encouraged to share links to the WeChat group established for their respective guidelines to expand the pool of potential respondents.

We implemented screening measures and excluded respondents who did not have relevant consensus experience or complete the questionnaire. Furthermore, we limited each participant’s WeChat account to a single questionnaire submission to prevent duplicates.

### Survey questionnaire

We drafted a preliminary questionnaire by sorting out consensus methodological literature and invited clinical experts and methodologists to revise and improve. The questionnaire created a separate section for guideline chairs, working group members, consensus panelists, and reviewers. This survey allows respondents with multiple guideline development roles to complete their respective sections. The questionnaire had three main domains: demographic information, consensus experience, and knowledge of consensus methods. All participants completed the “Basic Information” section, which includes questions such as gender, age, job title, professional area, and the number of previous guidelines involved (1–13). Questions 14–33 were intended for guideline chairs, 34–54 for working group members, 55–74 for panels, and 75–80 for reviewers.Within each role-specific section, questions were a combination of open-ended and multiple-choice questions, with specific details available in Supplementary File 1. The questionnaire underwent a pilot testing phase to ensure its effectiveness and clarity. During this phase, three participants from each role category completed the questionnaire, providing feedback on its comprehensibility and relevance. Adjustments were made based on their input to improve the questionnaire’s quality and suitability for the study. Expert reviews and a pre-survey investigation assessed surface and content validity. Reliability testing was not conducted because most questions were not scored.

The consensus experience section included questions such as the method used to reach consensus, the involvement of methodologists, and the adequacy of opinion expressed. Respondents were also asked about difficulties and challenges, candidates to host the meeting, and other factors influencing consensus. The knowledge section included questions on factors that should not be considered when making recommendations, conditions for making a strong recommendation and strategies for presenting health economic evidence during the development of recommendations.

### Data collection

This survey utilised the Wenjuanxing Online Survey Service (www.wjx.cn), a platform developed by Changsha Ranxing Technology in Shanghai, China, similar to SurveyMonkey. The collected data were exported to the statistical software program SPSS® 24 for data verification and analysis.

### Statistical analyses

The responses to the questionnaire were analysed and reported using descriptive statistics, including the mean, standard deviation, and percentage. The results were described using the median and interquartile range for data that were not normally distributed. Qualitative data are summarised and collated. The analysis primarily focused on understanding the consensus methods, adequacy of opinion from different roles and severity of factors affecting consensus results.

## Results

### Demographic characteristics

A total of 2,426 people clicked on the survey link, but only 290 of them completed the questionnaire, with a balanced gender distribution of 134 females (46.21%) and 156 males (53.79%). The participants came from 31 provinces or municipalities across China, with the most significant proportion being Beijing (107, 36.9%). Most participants (211, 72.76%) held senior professional titles, and 186 (64.14%) reported holding an ongoing guideline project. The participants included guideline chairs (71, 24.48%), consensus panel members (162, 46.9%), working group members (136, 55.86%), and reviewers (63, 21.72%).Among them, the most prominent background was TCM clinicians (150, 51.72%). (Table 1).

**Table 1:** Demographic characteristics of the study participants (n = 290)

Demographics	Distribution of responses	
	n	%
Gender		
Female	134	46.21
Male	156	53.79
**Age**		
18–40	97	33.45
41–60	178	61.38
≥ 61	15	5.17
**Professional title**		
Senior title	211	72.76
Intermediate title	46	15.86
Elementary Title	12	4.14
Postgraduate	16	5.52
others	5	1.72
**Ongoing guidelines**		
Yes	186	64.14
No	104	35.86
**Background**		
TCM clinicians	150	51.72
Integrative Medicine clinicians	32	11.03
Western medicine clinicians	21	7.24
Methodologists	39	13.45
Statisticians	2	0.69
Pharmacy specialists	5	1.72
Staff of academic societies	12	4.14
Others	29	10
**The role in development of the guideline**		
Guideline chair	71	24.48
Guideline panel	136(95)^^^	46.9
Working group	162(146)^^^	55.86
Reviewers	63	21.72
**Number of guidelines the respondents involved**		
0	1	0.34
<3	154	53.1
3–5	69	23.79
>5	66	22.76
**Guidelines of the respondents reviewed**		
0	99	34.14
<3	89	30.69
3–5	60	20.69
>5	42	14.48
**Paradigm of clinical guidelines**		
Textbook style*	34	11.72
Specific clinical problem-oriented guidelines	250	86.21
Others	6	2.07

*:Textbook style: textbook-like, with background, definitions, considerations for therapeutic diagnosis, preventive care, etc

^:This question is multiple-choice. While 136 individuals claimed to be panel members, only 95 completed the panel section. Similarly, out of the 162 individuals with previous work group experience, only 146 completed the working group section

### Guideline chairs’ experiences (n = 71)

The guideline chairs most commonly used the Consensus Development Conference (CDC) (n = 28, 39.44%) and the Delphi method (n = 23, 32.39%) to make recommendations (Supplementary file 2). Most guideline chairs only had a preliminary understanding of consensus methods (n = 34, 47.89%). Guideline chairs consulted with methodologists to determine a consensus approach (n = 23, 32.39%). Over half of the chairs (n = 63, 88.73%) considered face-to-face meetings necessary. The guideline chairs noted that the credibility of the consensus was affected by adequate retrieval of evidence. The most common problem was that the panel usually failed to respond promptly to the Delphi survey (Supplementary file 3).

### Panel members’ experiences (n = 95)

According to the survey, 47.37% (n = 45) of panel members believed that the guideline chairs should be responsible for presiding over the consensus meetings. Only 38.95% (n = 37) of the panels had training experience in consensus methodology. During the consensus process, the panel members encountered several typical situations, including experts being poor listeners (n = 43, 45.26%), indecipherable materials sorted out by the working group (n = 40, 42.11%), and poor communication due to different backgrounds (n = 40, 42.11%)(Supplementary file 4). On average, the panel members reported a severity score of 7.51 (ranging from 0 to 10) for feeling oppressed during the consensus process.

### Working group members’ experiences (n = 146)

**Fig. 1:**
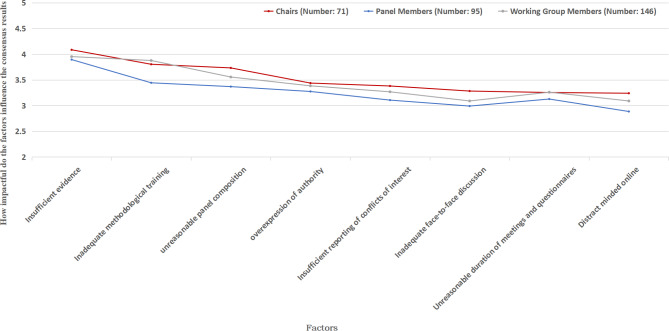
Different roles’ views on the degree of influence of factors on the results

The Delphi method was the most commonly used method for making recommendations among the working group members (n = 68, 46.58%). However, most participants only had a preliminary understanding of consensus methods (n = 66, 45.21%). The most common way to learn about the consensus methods was through conference reports (n = 94, 64.38%). Working groups often decide which consensus method to use (n = 61, 41.78%). The working group members identified three adverse factors that affected the consensus most seriously: insufficient relevant evidence (MD = 3.95), inadequate methodological training (MD = 3.88), and unreasonable panel composition (MD = 3.55) (Fig. 1). The most common challenges working groups encounter during the consensus process are conflicts between experts (n = 85,58.22%) and ineffective communication due to different backgrounds (n = 76,52.05%) (Supplementary file 5).

### Reviewers’ experiences (n = 63)

The design of questions for reviewers differs from the other three roles and does not pertain to the questions mentioned above. Reviewers rated high-quality guidelines at 8.36 ± 0.93 points (out of 10) and low-quality guidelines at 5.71 ± 2.13 points, with an overall average of 7.21 ± 1.31 points. Reviewers identified deficiencies in the consensus process, including clinical experts’ limited understanding of consensus methods and the resulting recommendations failing to guide clinical practice. Recommendations for improving consensus implementation require the involvement of multi-expertise experts, enhancing quality control of the consensus process, and intensifying training on methodology.

### Knowledge of guideline development methodology (n = 290)

The questionnaire set four questions about the methodology of guideline development. Among the participants, a total of 178 individuals (61.38%) demonstrated accurate judgment in selecting the appropriate option when faced with a range of clinical questions, distinguishing between those that were overly broad and excessively narrow. In the evidence-to-recommendations (EtD) framework [15] question, 148 participants (51.03%) selected all key factors that determine the strength of a recommendation. Only 23 participants (7.93%) correctly answered the multiple-choice question: Which scenarios can strong recommendations be made? Furthermore, 132 participants (45.52%) considered that each recommendation should include economic considerations when making recommendations.

### Overall experiences of all respondents (n = 312)

**Fig. 2:**
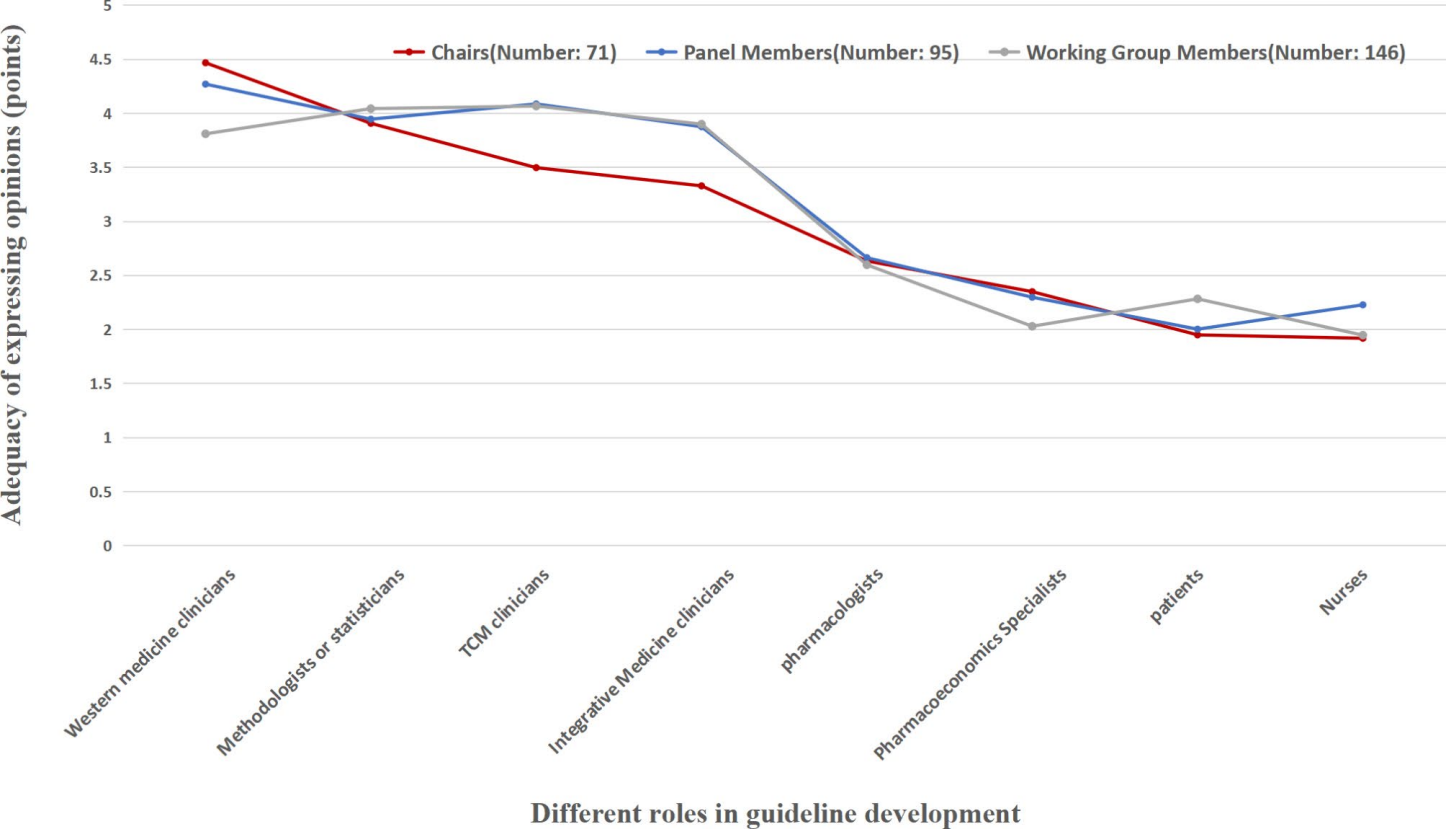
Different roles’ perceptions of the adequacy of each role’s involvement in the consensus process

The opinions of the chair, panel and working group are nearly the same regarding the influence of factors on the consensus result (Fig. 1). Nevertheless, when considering these factors, it was observed that chairs, compared to working groups and panels, considered these factors to significantly influence consensus results. Regarding the adequacy of involvement, the three roles generally felt that participation by pharmacoeconomists, patients, and nurses was insufficient (Fig. 2). A total of 209 respondents (66.99%) agreed, while 51 (16.35%) strongly agreed with the idea of establishing multiple chairs within the panel. However, there were dissenting opinions from some chairs (n = 11, 15.49%) who disagreed or strongly disagreed with the notion, which diverged from the perspectives of the working group (n = 9, 6.16%) and panel (n = 6, 6.32%). Furthermore, a significant majority of the 295 respondents (83.34%) agreed or strongly agreed with the proposition of examining experts’ background knowledge and willingness before their inclusion in the panel. The Delphi method emerged as the most frequently utilised consensus approach (n = 132, 42.31%); however, the respondents only had a preliminary understanding (n = 147, 47.12%). Additionally, an overwhelming 91.35% (n = 285) of the respondents believed that a face-to-face consensus discussion should be mandatory during the consensus process.

## Discussion

### Summary of findings

Our research focused on the roles involved in developing guidelines in China, including guideline chairs, working group members, panelists, and reviewers. We found that while these individuals may have limited knowledge about the consensus methodology, they are eager to learn and improve their understanding of it. Unfortunately, there is an imbalance in the composition of consensus groups, which limits the representation of different perspectives. However, guideline developers recognise the importance of incorporating a complexity perspective. We also identified several challenges during the consensus process, including experts’ inability to respond to questionnaires in a timely manner, inadequate preparation of consensus evidence materials, insufficient training in guideline development methodology, and ineffective communication among experts from different knowledge backgrounds. Despite these challenges, participants supported certain methodological viewpoints, such as establishing a multigroup system and conducting pre-enrollment surveys for panel members.

### Strengths and limitations

The survey pioneers a comprehensive exploration by delving into participants’ attitudes, experiences, and emotions and identifying challenges and difficulties encountered in applying consensus methods of guideline development. This survey, led by the Chinese Association of Chinese Medicine (CACM), is the most authoritative academic association in the field of TCM in China. The questionnaire categorises participants into guideline chairs, working group members, panelists, and reviewers, aligning with the existing characteristics of guideline development in China [14]. The survey utilises purposive rather than random sampling, enhancing the sample’s representativeness. However, sharing the survey link only within WeChat groups may have reduced its visibility and potential responses. Nevertheless, the precision of the results may be influenced by selection bias and recall bias.

### Comparison with previous research

We found no articles exploring participants’ experiences, attitudes, and emotions in implementing the consensus method in guidelines. Previous studies on consensus methods are often systematic reviews [14, 16–18] and theoretical discussions [19, 20]. These four systematic reviews provided comprehensive appraisals of the application of consensus methods in TCM guidelines [14], Delphi research [16, 17], and medical education research [18]. However, there were deviations in the application processes of the consensus method among these studies, and the reporting quality needed to be improved. Stefano [21] found that the modified Delphi method is more suitable for guideline development through large-scale meeting discussions, which coincides with our findings that respondents expect face-to-face meetings rather than just questionnaires.

### Implications for future research

Previous research on applying consensus methods in guidelines has predominantly relied on literature-based studies. However, the consensus process involves interpersonal discussion and interaction, and the emotions and behaviours of participants cannot be adequately captured through literature alone. If our goal is to harness the advantages of consensus and incorporate a complexity perspective to develop better and more practical recommendations, our study proposes two research recommendations. First, we suggest conducting field surveys or participant observations to investigate what occurs during the consensus process and gain insights into the advantages and barriers involved. Second, a manual for the consensus process should be developed to provide better guidance on specific issues, such as preventing excessive authority interference and improving interaction. We recommend conducting more qualitative research to enhance the quality of consensus and strengthen the reliability of guidelines.

### Implications for consensus method in guideline development

In clinical practice guideline development, the experience and cooperation of different roles throughout the consensus process are critical factors in achieving substantial results. Therefore, we propose the following:

#### Organising training in methodology conscientiously

Survey results showed a positive attitude towards methodological training among participants. However, only 55.86% of the respondents received consensus method training, and only 46.9% received EtD [22] training, indicating a limited understanding of the method. This deficiency can significantly affect the quality of the final recommendations. Thus, we suggest that guideline leaders, working groups, and panels receive rigorous methodological training on ① PICO issues [23]; ② systematic review and meta-analysis; ③ the GRADE [22] system; and ④ consensus methods to ensure high-quality guidelines.

#### Increase the enthusiasm of the panel

The willingness and enthusiasm of experts to express their opinions are essential to achieving a consensus, whether it is a questionnaire survey or a face-to-face meeting. The most significant drawback of a questionnaire survey is its low response rate. Thus, it is important not to complicate the questionnaire, which may decrease participation enthusiasm. To increase participant enthusiasm, ensuring their interest in the topic and understanding of their roles as research partners is crucial. Studies have shown [24] that the prompt collection and organisation of expert opinions can enhance experts’ willingness to participate. A previous study [25] suggested that consistent communication and reminders enable more effective feedback throughout the consensus process. Finally, obtaining informed consent from participants and explaining the study’s significance and the effort required to participate is essential.

#### A multichair system should be established in the panel

The research results indicate that nurses, patients, and health economists may need support to fully express their opinions in the consensus process due to the dominance of authoritative clinicians. However, it is critical to consider multiple viewpoints to ensure that guideline recommendations are feasible, practical, and cost-effective. We suggest promoting public participation in consensus discussions by assembling a patient group and electing patient representatives to participate in the consensus. We suggest implementing a multichair system to enhance the diversity of participation and amplify the voices of underrepresented groups.

#### Enhancing the consensus process through multi-expertise experts

Bringing together stakeholders with different perspectives and facilitating consensus is quite challenging, which is why experts with multi-expertise are crucial to the process.These individuals possess specialized clinical knowledge and expertise in methodology, enabling them to foster collaboration and communication among stakeholders with varying perspectives. They also shoulder the responsibility of quality control, proactively monitoring and evaluating the consensus process to ensure its integrity and validity.

Multi-expertise experts play a pivotal role in ensuring that consensus outcomes are aligned with the real-world needs of clinical practice. They bridge the gap between research and practical application by possessing expertise in multiple interventions, ideally covering all interventions in the comparison. By generating evidence-based, practical, and contextually relevant recommendations, they empower healthcare professionals and patients to make well-informed decisions that lead to improved outcomes.

#### The consensus method in TCM guideline development

One distinctive feature of TCM guideline development is the composition of the consensus panel, which sets it apart from Western medicine guidelines. TCM consensus panels typically encompass TCM physicians, Western medicine physicians, and occasionally integrative medicine physicians. However, there is no consensus on the precise ratio of physicians from each group required for optimal composition.

The emphasis in TCM on syndrome differentiation and treatment can introduce complexity into clinical scenarios. Consequently, we recommend a diverse panel composition that includes a certain number of Western medicine experts, ensuring representation from each relevant department. This approach acknowledges the inherent differences in perspectives and understanding between TCM and Western medicine practitioners.

The unique blend of TCM and Western medicine perspectives underscores the importance of consensus in TCM guideline development. To facilitate effective communication and mitigate potential survey misunderstandings among panel members with varying viewpoints, we recommend using the nominal group method rather than the Delphi method. This approach aligns with the multifaceted nature of TCM guideline development, promoting collaboration and consensus-building among experts from diverse backgrounds.

## Conclusion

The participants involved in developing TCM guidelines, including guideline chairs, panel members, working group members, and reviewers, attach a positive inclination towards methodological learning. However, there are also problems, such as insufficient patient participation and ineffective communication between experts. In the future, methodologists should strengthen methodological training for participants and develop a standardised consensus process and quality assessment tools to improve the quality of guidelines.

### Electronic supplementary material

Below is the link to the electronic supplementary material.

Supplementary Material 1

Supplementary Material 2

Supplementary Material 3

Supplementary Material 4

Supplementary Material 5

## Data Availability

Data associated with this study will be available upon request from corresponding author.

## Abbreviations

NGT: Nominal group technique
TCM: Traditional Chinese Medicine
CACM: China Association of Chinese Medicine
HREC: Human Research Ethics Committee
CPGs: clinical practice guidelines
EtD: evidence-to-recommendations
PICO: Population, Intervention, Comparison and Outcome
GRADE: Grade of Recommendations Assessment, Development and Evaluation
RCTs: Randomized Controlled Trials

## Declarations

### Provenance and peer review

Not commissioned; externally peer reviewed.
