# Supplementary material for: How well did the consensus methods apply in the guideline development of traditional Chinese medicine: a web-based survey in China
Source: BMC Med Res Methodol. 2023 Nov 10;23:264. doi: 10.1186/s12874-023-02087-0 (PMC10636859; doi:10.1186/s12874-023-02087-0)
Supplement: Supplementary file 1 — Supplementary Material 1 [file 12874_2023_2087_MOESM1_ESM.docx]

**How did your guidelines achieve expert consensus?**

Dear Expert:

Hello! We are the research team from the Center for Evidence-Based Medicine, Beijing University of Traditional Chinese Medicine. We carried out this survey to promote the expert consensus method in guidelines development, improve its practicality and efficiency, understand the difficulties and deficiencies of this method, and abundanty find its influencing factors and technical parameters in the field of traditional Chinese medicine. We hope your active participation and cooperation. There are some open questions in this questionnaire. Through this questionnaire , we hope to know the current situation and problems of the consensus method in guidelines development. Thank you a lot for filling it out careabundanty!

Consensus Method Research Group
Beijing University of Traditional Chinese Medicine

October 2021

Instructions for filling out the questionnaire:
1. We will keep your personal information filled in in this survey strictly confidential. All answers will only be used for statistical analysis. All survey data collation personnel will strictly abide by the confidentiality agreement. If you have **a abundant understanding of the above content and agree to participate in this questionnaire survey**, please fill out it.

Part 1: Basic Information

1. Have you chaired, participated, directed or reviewed a guideline or expert consensus? [Single choice] *

| ○ yes |
| --- |
| ○Never participated at all (please skip to the end of the questionnaire and submit)  In order to make our research more comprehensively, we hope you can help forward it to your colleagues, classmates and experts who you think have led/participated in the expert consensus process in the guidelines or expert consensus. We will be very grateful if you are willing to forward! |
| ○I have already filled out the questionnaire (please skip to the end of the questionnaire and submit) |

2. Your gender [Single choice] *

| ○Male |
| --- |
| ○ Female |

3. Your age [fill in the blank] *

__________________________________

4. Your job title [Single choice] *

| ○Advanced |
| --- |
| ○Intermediate |
| ○Elementary |
| ○Graduate student |
| ○Other_________________ |
| ○ Not applicable |

5. Your professional field (such as respiratory, oncology, health economics or other fields) [fill in the blank] *

__________________________________

6. How many times have you led guidelines or expert consensus development? [Single choice] *

| ○0 times |
| --- |
| ○ Less than 3 times |
| ○3-5 times |
| ○ More than 5 times |

7. How many times have you participated in the guidelines or expert consensus development? [Single choice] *

| ○0 times |
| --- |
| ○ Less than 3 times |
| ○3-5 times |
| ○ More than 5 times |

8. How many times have you reviewed exposure drafts for guidelines or expert consensus? [Single choice] *

| ○0 times |
| --- |
| ○ Less than 3 times |
| ○3-5 times |
| ○ More than 5 times |

9. Do you have any ongoing guideline or expert consensus project? [Single choice] *

| ○ yes |
| --- |
| ○ no |

10. Your main business status when you lead/participate in the guideline development? [Single choice] *

| ○ Traditional Chinese Medicine Physician |
| --- |
| ○Western Medicine Physician |
| ○ Physician integrating traditional Chinese and Western medicine |
| ○ Nurse |
| ○Methodologist |
| ○Statistician |
| ○Pharmaceutical Economist |
| ○Pharmacy expert |
| ○Member of professional society |
| ○Patient |
| ○abundant-time graduate student |
| ○Other_________________ |

11. Which of the following areas are you good at, or have you received systematic training? [multiple choice] *

| □Clinical knowledge related to guidelines |
| --- |
| □How to raise clinical questions (PICO model) |
| □Relevant knowledge of systematic review |
| □Evidence rating method |
| □ Relevant methods for forming guideline recommendations |
| □Expert consensus method |
| □Other_________________ |

12. What paradigm do you think clinical practice guidelines should have? [Single choice] *

| ○Textbook style (similar to textbook style, with background, definition, precautions for treatment and diagnosis, prevention, etc.) |
| --- |
| ○Problem-based style (the clinical practice guideline should revolve around PICO problems (prospect problems, that is, with the four elements of patients, interventions, controls, and outcomes) that need to be solved first. The guideline should have evidence that has been systematically sorted out and strictly evaluated, considering multiple factors, and finally make a recommendation) |
| ○Others, please add_________________ |

13. Which of the following roles fits your role in guideline development? [multiple choice] *

| □Leader for guideline development |
| --- |
| □Working group members (ensure the normal operation of guideline development and organize staffs who have participated in the expert consensus method) |
| □Members of the expert group (identify clinical problems and form recommendations in the guideline) |
| □Guideline review expert (responsible for the standardization of the guideline draft and propose revision opinions) |

**Part II: The Application of Consensus Methods**

The second part is divided into 4 sets of questions by role. Please fill in according to your own role. If you choose 2 or more roles, you are also welcome to fill in the questionnaire for multiple roles. Thank you for your support.

**As the leader of the guideline:** please recall the process of **the latest** guideline or expert consensus, and answer the following questions.

14. What was the expert consensus approach you used to form recommendations in the most recent guideline you led? [Single choice] *

| ○Delphi method |
| --- |
| ○ Modified Delphi method |
| ○Nominal group technique |
| ○ Consensus meeting method |
| ○Other_________________ |
| ○Can’t tell clearly (can briefly describe) _________________ |

15. Is the identification of the clinical question consistent with the consensus approach used to form the recommendation? [Single choice] *

| ○ Inconsistent |
| --- |
| ○ Consistent |
| ○ Can't remember |

16. How well do you know the consensus method used? [Single choice] *

| ○Do not understand |
| --- |
| ○ Understand |
| ○ Familiar |
| ○ Master |

17. How did you learn about this consensus method? [Multiple choice] *

| □Domestic related literature |
| --- |
| □Foreign related literature |
| □Participate in targeted training |
| □Listen to reports from members and meeting reports, or participate in reviews |
| □Other_________________ |

18. How did you choose the above consensus method? [Single choice] *

| ○Determined by the leader |
| --- |
| ○Determined by the working group |
| ○Determined by the leader after proposed by the working group |
| ○Determined after consulting methodologist |
| ○ I don’t know how to choose |
| ○Other_________________ |

19. During the selection process of the consensus method, the degree of participation of methodological experts **(most recently)** [Single choice] *

| not involved at all | ○1 | ○2 | ○3 | ○4 | ○5 | completely dominant |
| --- | --- | --- | --- | --- | --- | --- |

20. Do you think a face-to-face meeting is necessary in the consensus process? Why? (You can briefly state the reason on the transverse line) [Single choice] *

| ○Very unnecessary_________________ |
| --- |
| ○It is not necessary__________________ |
| ○It doesn’t matter__________________ |
| ○It is necessary to _________________ |
| ○It is very necessary to__________________ |

21. Who do you think should lead the process of the consensus meeting? Why? (You can briefly state the reason on the transverse line) [Single choice] *

| ○Guideline leader _________________ |
| --- |
| ○Core members of the working group_________________ |
| ○ Methodologist _________________ |
| ○Other_________________ |

22. Do you agree that the expert group should determine the team leaders and their responsibilities from different identities? (such as clinical team leader, methodological team leader, patient team leader) (You can briefly state the reason on the transverse line) [Single choice] *

| ○Strongly Disagree_________________ |
| --- |
| ○ Disagree _________________ |
| ○It doesn’t matter__________________ |
| ○ Agree _________________ |
| ○Strongly Agree_________________ |

23. Do you agree that when setting up an expert group, we should conduct a thorough survey of the experts to understand their knowledge background, cognitive prejudices and enthusiasm for participation? (You can briefly state the reason on the transverse line) [Single choice] *

| ○Strongly Disagree_________________ |
| --- |
| ○ Disagree _________________ |
| ○It doesn’t matter__________________ |
| ○ Agree _________________ |
| ○Strongly Agree_________________ |

24. During the guideline or expert consensus development you recently led, did all participants abundanty express their opinions? Please rate the extent to experts in each capacity voiced their views. [Matrix Single choice Questions] *

|  | None of the roles participated | Very insufficient | insufficient | have no idea | abundant | very abundant |
| --- | --- | --- | --- | --- | --- | --- |
| Traditional Chinese Medicine Physician | ○ | ○ | ○ | ○ | ○ | ○ |
| Western medicine physician | ○ | ○ | ○ | ○ | ○ | ○ |
| Physician integrating traditional Chinese and Western medicine | ○ | ○ | ○ | ○ | ○ | ○ |
| Nurse | ○ | ○ | ○ | ○ | ○ | ○ |
| Methodologist or statistician | ○ | ○ | ○ | ○ | ○ | ○ |
| pharmaceutical economist | ○ | ○ | ○ | ○ | ○ | ○ |
| Pharmacy expert | ○ | ○ | ○ | ○ | ○ | ○ |
| patient | ○ | ○ | ○ | ○ | ○ | ○ |

25. Please judge how serious the following negative aspects on the consensus process is? (Scored on a scale of 1-5. 1 means no impact, 5 means very serious impact) [matrix scale question] *

|  | No effect | slightly affected | influential | Serious impact | Very serious impact |
| --- | --- | --- | --- | --- | --- |
| Inadequate retrieval and evidence presentation | ○ | ○ | ○ | ○ | ○ |
| The length of the meeting and the questionnaire are unreasonable | ○ | ○ | ○ | ○ | ○ |
| There are experts absent from the meeting | ○ | ○ | ○ | ○ | ○ |
| Overexpression of the authority or powerful figure | ○ | ○ | ○ | ○ | ○ |
| Inadequate reporting of conflicts of interest and lax management | ○ | ○ | ○ | ○ | ○ |
| Insufficient methodological training prior to undertaking the guideline work | ○ | ○ | ○ | ○ | ○ |
| Experts are easily distracted in online consensus meetings | ○ | ○ | ○ | ○ | ○ |
| Insufficient discussions during face-to-face meetings | ○ | ○ | ○ | ○ | ○ |
| The composition of the expert group is unreasonable | ○ | ○ | ○ | ○ | ○ |
| Publish the expert opinions with real names or let the experts express their opinions in public without notification | ○ | ○ | ○ | ○ | ○ |

26. In addition to the content mentioned above, do you have any other additions to the bad aspects that affect the consensus process? [fill in the blank]

__________________________________

27. Have you encountered any of the following situations during the consensus process? [Multiple choice] *

| □Due to the different knowledge backgrounds of different experts, invalid communication happened in the consensus meeting |
| --- |
| □Some experts are not good at listening to others' opinions |
| □Some experts did not respond to the questionnaire in time |
| □Consensus cannot be reached after many rounds of discussions |
| □Contradictions between non-medical and medical roles in the expert group, such as methodologists, health economists, patients, etc. |
| □Contradictions between medical roles in different fields, such as traditional Chinese medicine, western medicine, rehabilitation, nursing, nutrition, etc. |
| □Consensus panel experts quit the panel due to dissatisfaction with the consensus process or results |
| □ The consensus process is awkward and difficult to maintain |
| □In other cases, please add _________________ |

28. What do you think the proportion of people with evidence-based medicine background to the total working group should be?
____________% [fill in the blank] *

29. Do you agree to train the consensus group experts on the basic knowledge of guideline development methodology before the consensus process starts? [Single choice] *

| ○Strongly disagree |
| --- |
| ○ Disagree |
| ○ Does not matter |
| ○ Agree |
| ○ Strongly agree |

30.What do you think is **the business area** that you most need to seek help from during the guideline development process? [Multiple choice] *

| □Evidence-based medical knowledge for guideline development_________________ |
| --- |
| □Clinically relevant development_________________ |
| □Clinical related knowledge_________________ |
| □I don’t feel powerless__________________ |
| □Other_________________ |

31. As the leader of the guideline, what issues are you most concerned about during the expert consensus process? [fill in the blank]

__________________________________

32. As the leader of the guideline, what is the most embarrassing thing for you in the process of expert consensus? [fill in the blank]

__________________________________

**Part II: Survey on the Application of Consensus Methods**

**As a core member of the working group** : Please recall **the latest** guideline or expert consensus formulation process and answer the following questions.

33. What was the expert consensus approach you used to form recommendations in the most recent lead guideline? [Single choice] *

| ○Delphi method |
| --- |
| ○Nominal group technique |
| ○ Modified Delphi method |
| ○ Consensus meeting method |
| ○Other_________________ |
| ○Can’t tell clearly (can briefly describe) _________________ |

34. Is the identification of the clinical question consistent with the consensus approach used to form the recommendation? [Single choice] *

| ○ Inconsistent |
| --- |
| ○ Consistent |
| ○ can't remember |

35. How well do you know the consensus method adopted? [Single choice] *

| ○ do not understand |
| --- |
| ○ understand |
| ○ Familiar with |
| ○Master |

36. How did you know about this consensus method? [Single choice] *

| □Domestic related literature |
| --- |
| □Foreign related literature |
| □Participate in targeted training |
| □Listen to reports from staff or leaders, meeting reports or participate in reviews |
| □Other_________________ |

37. How did you choose the expert consensus method in the latest guideline you participated in? [Single choice] *

| ○ proposed by the guideline leader |
| --- |
| ○Decided by working group discussion |
| ○Determined by the leader after proposed by the working group |
| ○Determined after consultation with a methodologist |
| ○Other_________________ |

38. What are the difficulties and challenges you have encountered in the process of implementing the consensus method? [Single choice] *

| □Consensus-related material preparation |
| --- |
| □Tacit cooperation between working groups |
| □Data statistics of expert opinion voting |
| □Writing of late meeting minutes and consensus process |
| □ During the development of international guidelines, experts have problems with time difference |
| □The logic of the language reply content of expert opinions is not clear, making it difficult to understand the real meaning of experts |
| □In the stage of collecting and identifying clinical problems, some experts do not understand the concept of PICO |
| □The experts did not respond to the questionnaire in time |
| □Consensus cannot be reached after many rounds of discussions |
| □Contradictions between non-medical and medical roles in the expert group, such as methodologists, health economists, patients, etc. |
| □Contradictions between different medical roles, such as traditional Chinese medicine, western medicine, rehabilitation, nursing, nutrition, etc. |
| □Other_________________ |

39. Do you think a face-to-face meeting is necessary in the consensus process? Why? (You can briefly state the reason in the horizontal line) [Single choice] *

| ○Very unnecessary_________________ |
| --- |
| ○It is not necessary__________________ |
| ○It doesn’t matter__________________ |
| ○It is necessary to _________________ |
| ○It is very necessary to__________________ |

40. Who do you think should lead the process of the consensus meeting? Why? (You can briefly state the reason in the horizontal line) [Single choice] *

| ○Guide lead _________________ |
| --- |
| ○Core members of the working group_________________ |
| ○ Methodologist _________________ |
| ○Other_________________ |

41. Do you agree that the expert group is divided into groups according to different identities (such as clinical group, methodological group, and patient group) and the group leader is set as the representative of the group members? You can briefly explain the reasons horizontally [Single choice] *

| ○Strongly Disagree_________________ |
| --- |
| ○ Disagree with _________________ |
| ○It doesn’t matter__________________ |
| ○ Agree to _________________ |
| ○Strongly Agree_________________ |

42. Do you agree that when setting up an expert group, we should conduct a thorough investigation of the experts to understand their knowledge background, cognitive prejudices and enthusiasm for participation? (You can briefly state the reasons in the horizontal line) [Single choice] *

| ○Strongly Disagree_________________ |
| --- |
| ○ Disagree with _________________ |
| ○It doesn’t matter__________________ |
| ○ Agree to _________________ |
| ○Strongly Agree_________________ |

43. During your most recent guideline expert consensus process, were all participants abundanty voiced? Please rate the extent to which experts in each practice capacity abundanty voiced their views. [Matrix Single choice Questions] *

|  | None of the roles participated | very inadequate | insufficient | have no idea | abundant | very abundant |
| --- | --- | --- | --- | --- | --- | --- |
| Traditional Chinese Medicine Physician | ○ | ○ | ○ | ○ | ○ | ○ |
| Western medicine physician | ○ | ○ | ○ | ○ | ○ | ○ |
| Integrative Chinese and Western Medicine Physician | ○ | ○ | ○ | ○ | ○ | ○ |
| Nurse | ○ | ○ | ○ | ○ | ○ | ○ |
| Methodologist or statistician | ○ | ○ | ○ | ○ | ○ | ○ |
| pharmaceutical economist | ○ | ○ | ○ | ○ | ○ | ○ |
| Pharmacy expert | ○ | ○ | ○ | ○ | ○ | ○ |
| patient | ○ | ○ | ○ | ○ | ○ | ○ |

44. Have you ever encountered any of the following situations during the consensus process? [Single choice] *

| □Due to the different knowledge backgrounds of different experts, encountered invalid communication in the consensus meeting |
| --- |
| □Some experts are not good at listening to others' opinions |
| □The experts did not respond to the questionnaire in time |
| □Consensus cannot be reached after many rounds of discussions |
| □Contradictions between non-medical and medical roles in the expert group, such as methodologists, health economists, patients, etc. |
| □Contradictions between different medical roles, such as traditional Chinese medicine, western medicine, rehabilitation, nursing, nutrition, etc. |
| □Consensus panel experts quit the panel due to dissatisfaction with the consensus process or results |
| □ The consensus process is awkward and difficult to maintain |
| □In other cases, please add _________________ |

45. Please judge how serious the impact of the following negative aspects on the consensus process is? (Scored on a scale of 1-5, 1 means no impact, 5 means very serious impact) [matrix scale question] *

|  | No effect | slightly affected | influential | Serious impact | The impact is very serious |
| --- | --- | --- | --- | --- | --- |
| Insufficient search and presentation of relevant evidence | ○ | ○ | ○ | ○ | ○ |
| The length of the meeting and the length of the questionnaire are unreasonable | ○ | ○ | ○ | ○ | ○ |
| There are experts absent from the meeting | ○ | ○ | ○ | ○ | ○ |
| Overexpression of an authority or powerful figure | ○ | ○ | ○ | ○ | ○ |
| Inadequate reporting and lax management of conflicts of interest | ○ | ○ | ○ | ○ | ○ |
| Insufficient methodological training prior to undertaking guideline work | ○ | ○ | ○ | ○ | ○ |
| Experts are easily distracted in online consensus meetings | ○ | ○ | ○ | ○ | ○ |
| Insufficient discussions during face-to-face meetings | ○ | ○ | ○ | ○ | ○ |
| The composition of the expert group is unreasonable | ○ | ○ | ○ | ○ | ○ |
| Under the premise of not knowing the experts, publish the expert opinions with real names or let the experts express their opinions in public | ○ | ○ | ○ | ○ | ○ |

46. In addition to the content mentioned above, do you have any other additions to the bad aspects that affect the consensus process? [fill in the blank]

__________________________________

47. What do you think is the proportion of people with evidence-based medicine background in the working group to the total working group?
____________% [fill in the blank] *

48. Do you agree to train consensus group experts on the basic knowledge of guideline development methodology before the consensus process starts? [Single choice] *

| ○ strongly disagree |
| --- |
| ○ disagree |
| ○ does not matter |
| ○ Agree |
| ○ strongly agree |

49. How much do you think methodologists should be involved in designing the entire consensus process? [Single choice] *

| completely unnecessary | ○1 | ○2 | ○3 | ○4 | ○5 | completely dominant |
| --- | --- | --- | --- | --- | --- | --- |

**area of your business** during the entire guideline development process ? Reasons can be briefly stated in the horizontal line. [Single choice] *

| □Evidence-based medical knowledge for guideline development_________________ |
| --- |
| □Clinically relevant development_________________ |
| □Clinical related knowledge_________________ |
| □Other_________________ |

51. As a member of the working group of the guideline, what concerns you most during the process of expert consensus? [fill in the blank]

__________________________________

52. As a member of the working group of the guideline, what is the most embarrassing problem for you in the process of expert consensus method? [fill in the blank]

__________________________________

**Part II: Survey on the Application of Consensus Methods**

**As a member of the consensus expert group** : Please recall **the last** guideline or expert consensus formulation process and answer the following questions

53. What was the expert consensus approach you used to form recommendations in the most recent lead guideline? [Single choice] *

| ○Delphi method |
| --- |
| ○Nominal group technique |
| ○ Modified Delphi method |
| ○ Consensus meeting method |
| ○Other_________________ |
| ○Can’t tell clearly (can briefly describe) _________________ |

54. Is the identification of the clinical question consistent with the consensus approach used to form the recommendation? [Single choice] *

| ○ Inconsistent |
| --- |
| ○ Consistent |
| ○ can't remember |

55. How well do you know the consensus method adopted? [Single choice] *

| ○ do not understand |
| --- |
| ○ understand |
| ○ Familiar with |
| ○Master |

56. How did you know about this consensus method? [Single choice] *

| □Domestic related literature |
| --- |
| □Foreign related literature |
| □Participate in targeted training |
| □Listen to staff reports, meeting reports or participate in reviews |
| □Other_________________ |

57. Do you think a discussion meeting is necessary in the consensus process? Why? (You can briefly state the reason in the horizontal line) [Single choice] *

| ○Very unnecessary_________________ |
| --- |
| ○It is not necessary__________________ |
| ○It doesn’t matter__________________ |
| ○It is necessary to _________________ |
| ○It is very necessary to__________________ |

58. Who do you think should lead the process of the consensus meeting? Why? (You can briefly state the reason in the horizontal line) [Single choice] *

| ○Guide lead _________________ |
| --- |
| ○Core members of the working group_________________ |
| ○ Methodologist _________________ |
| ○Other_________________ |

59. Do you agree that the expert group should determine the team leader and related responsibilities from different identities? (such as clinical team leader, methodological team leader, patient team leader) can briefly explain the reasons horizontally [Single choice] *

| ○Strongly Disagree_________________ |
| --- |
| ○ Disagree with _________________ |
| ○It doesn’t matter__________________ |
| ○ Agree to _________________ |
| ○Strongly Agree_________________ |

60. In the most recent guideline expert consensus process you participated in, were all participants abundanty voiced? Please rate the extent to which experts in each practice capacity abundanty voiced their views. [Matrix Single choice Questions] *

|  | None of the roles participated | very inadequate | insufficient | have no idea | abundant | very abundant |
| --- | --- | --- | --- | --- | --- | --- |
| Traditional Chinese Medicine Physician | ○ | ○ | ○ | ○ | ○ | ○ |
| Western medicine physician | ○ | ○ | ○ | ○ | ○ | ○ |
| Integrative Chinese and Western Medicine Physician | ○ | ○ | ○ | ○ | ○ | ○ |
| Nurse | ○ | ○ | ○ | ○ | ○ | ○ |
| Methodologist or statistician | ○ | ○ | ○ | ○ | ○ | ○ |
| pharmaceutical economist | ○ | ○ | ○ | ○ | ○ | ○ |
| Pharmacy expert | ○ | ○ | ○ | ○ | ○ | ○ |
| patient | ○ | ○ | ○ | ○ | ○ | ○ |

61. Have you encountered any of the following situations during the consensus process? [Single choice] *

| □ The relevant evidence presented by the working group is too numerous to read or understand |
| --- |
| □Due to the different knowledge backgrounds of different experts, encountered invalid communication in the consensus meeting |
| □Some experts are not good at listening to others' opinions |
| □ The search strategy and relevant evidence summary presented by the working group are not sufficient and cannot be used as a reference |
| □Your point of view is not heard or adopted |
| □Because there is no anonymity, I dare not express my opinion |
| □ There are obvious errors in the guideline recommendations, but they have not been corrected |
| □After participating, I feel that I am riding a tiger, but I am too embarrassed to quit |
| □ Withdrew due to dissatisfaction with the guideline development process or consensus results after participation |
| □Unable to respond to the questionnaire in time due to busy work and other reasons |
| □Consensus cannot be reached after many rounds of discussions |
| □Contradictions between non-medical and medical roles in the expert group, such as methodologists, health economists, patients, etc. |
| □Contradictions between different medical roles, such as traditional Chinese medicine, western medicine, rehabilitation, nursing, nutrition, etc. |

62. In addition to the above, do you have any other content to supplement? [fill in the blank]

__________________________________

63. Please judge how serious the impact of the following negative aspects on the consensus process is? (Scored on a scale of 1-5, 1 means no impact, 5 means very serious impact) [matrix scale question] *

|  | No effect | slightly affected | influential | Serious impact | The impact is very serious |
| --- | --- | --- | --- | --- | --- |
| Insufficient search and presentation of relevant evidence | ○ | ○ | ○ | ○ | ○ |
| The length of the meeting and the length of the questionnaire are unreasonable | ○ | ○ | ○ | ○ | ○ |
| There are experts absent from the meeting | ○ | ○ | ○ | ○ | ○ |
| Overexpression of an authority or powerful figure | ○ | ○ | ○ | ○ | ○ |
| Inadequate reporting and lax management of conflicts of interest | ○ | ○ | ○ | ○ | ○ |
| Insufficient methodological training prior to undertaking guideline work | ○ | ○ | ○ | ○ | ○ |
| Experts are easily distracted in online consensus meetings | ○ | ○ | ○ | ○ | ○ |
| Insufficient discussions during face-to-face meetings | ○ | ○ | ○ | ○ | ○ |
| The composition of the expert group is unreasonable | ○ | ○ | ○ | ○ | ○ |
| Under the premise of not knowing the experts, publish the expert opinions with real names or let the experts express their opinions in public | ○ | ○ | ○ | ○ | ○ |

64. In addition to the content mentioned above, do you have any other additions to the bad aspects that affect the consensus process? [fill in the blank]

__________________________________

65. Do you agree to train the members of the expert group on a unified guideline development methodology? [Single choice] *

| ○ strongly disagree |
| --- |
| ○ disagree |
| ○ does not matter |
| ○ Agree |
| ○ strongly agree |

66. Which issues need training or elaboration from the perspective of guideline development methods? [Single choice] *

| □The latest academic progress related to the topic of the guideline |
| --- |
| □Necessity and key methodological requirements for forming PICO questions |
| □Necessity and methodological points of optimal outcome indicators |
| □Key methodological points for forming the level of evidence |
| □ Key methodological requirements for forming strong and weak recommendations |
| □ Key technical requirements of the consensus process |
| □As an expert, the work and quality requirements that must be completed |
| □Other_________________ |

67. Do you agree that when setting up an expert group, we should conduct a thorough investigation of the experts to understand their knowledge background, cognitive prejudices and enthusiasm for participation? (You can briefly state the reason in the horizontal line) [Single choice] *

| ○Strongly Disagree_________________ |
| --- |
| ○ Disagree with _________________ |
| ○It doesn’t matter__________________ |
| ○ Agree to _________________ |
| ○Strongly Agree_________________ |

68. What do you think is the proportion of people with evidence-based medicine background in the working group to the total working group?
____________% [fill in the blank] *

69. How much do you think methodologists should be involved in designing the entire consensus process? [Single choice] *

| completely unnecessary | ○1 | ○2 | ○3 | ○4 | ○5 | completely dominant |
| --- | --- | --- | --- | --- | --- | --- |

**the business area that** you are most unable to do during the entire guideline development process ? Please specify in the horizontal line. [Single choice] *

| □Evidence-based medical knowledge for guideline development_________________ |
| --- |
| □Clinically relevant development_________________ |
| □Clinical related knowledge_________________ |
| □Other_________________ |

71. As a member of the expert group, what do you find most embarrassing in the process of expert consensus? [fill in the blank]

__________________________________

72. The last time you were a member of an expert group, how depressed were you during the expert consensus process? [Matrix text question] [Enter a number from 0 to 10] *

|  |  |
| --- | --- |
| scoring | ________________________ |

*After completing this question, please skip to question 79.

**Part II: Survey on the Application of Consensus Methods**

**As a guideline external review expert** : Please recall the last guideline or expert consensus formulation process and answer the following questions.

73. What do you think is the overall quality of the highest quality guide you have reviewed? [Matrix text question] [Enter a number from 0 to 10] *

|  |  |
| --- | --- |
| scoring | ________________________ |

74. How much do you think the overall quality of the lowest-quality guide you have reviewed should be rated? [Matrix text question] [Enter a number from 0 to 10] *

|  |  |
| --- | --- |
| scoring | ________________________ |

75. What do you think the average overall quality of the guidelines you reviewed should be rated as? [Matrix text question] [Enter a number from 0 to 10] *

|  |  |
| --- | --- |
| scoring | ________________________ |

76. What do you think is the weakest link of the guidelines you reviewed? [Fill in the blank]

__________________________________

77. What do you think is the biggest problem in the application of the current guideline expert consensus method? [fill in the blank]

__________________________________

78. Do you have any suggestions on how to better apply the expert consensus method in the guidelines? [fill in the blank]

__________________________________

The Third Part

79. Which of the following is not suitable as a clinical question for guideline development ? [Single choice] *

| □Is Chinese medicine suitable for hypertensive patients? |
| --- |
| □ How effective is Chinese herbal medicine in controlling blood pressure in hypertensive patients? |
| □Is it better to use integrated traditional Chinese and western medicine for hypertensive patients than to use conventional western medicine alone? |
| □For elderly hypertensive patients with coronary heart disease, is conventional treatment combined with traditional Chinese medicine more effective in avoiding the incidence and mortality of cardiovascular disease than conventional treatment alone? |
| □The above options are all suitable, and the reasons can be added_________________ |

should not be considered when forming recommendations ? [Single choice] *

| □Priority of issues |
| --- |
| □Certainty of evidence |
| □People's values |
| □ Demand for medical resources |
| □Fairness |
| □ Acceptability |
| □ Feasibility |
| □Clear opinions from authoritative experts |
| □ must be considered |

81. In which of the following situations can a strong recommendation or strong opposition be made based on extremely low-quality evidence? [Single choice] *

| □Although the quality of the evidence is very low, it is consistent with my general perception |
| --- |
| □ Uncertain benefit, definite harm |
| □ Potentially fair, with low risk or low cost |
| □Very sure of little benefit, possibly high risk or expensive |
| □It is extremely harmful |

82. In which way should health economics evaluation be presented when guidelines form recommendations? [Single choice] *

| ○ When forming each recommendation, take it into consideration |
| --- |
| ○ Presented separately as a separate section of the guide |
| ○ do not understand |

83. How did you hear about this questionnaire? [Single choice]

| ○ Issued directly by the researcher (WeChat invitation) |
| --- |
| ○ Issued directly by the researcher (email invitation) |
| ○ Learned by the Society (WeChat group invitation) |
| ○Issued by the society learned (email invitation) |
| ○Forwarded by friends, classmates or colleagues |
| ○Other_________________ |

84. Any answer from you is very meaningful to us. If you are interested in our research and willing to participate in the follow-up interview, I hope you can leave your name and contact information, thank you. (optional) [matrix text questions]

|  |  |
| --- | --- |
| Your Name | ________________________ |
| your contact information | ________________________ |
