# Supplementary material for: How well did the consensus methods apply in the guideline development of traditional Chinese medicine: a web-based survey in China
Source: BMC Med Res Methodol. 2023 Nov 10;23:264. doi: 10.1186/s12874-023-02087-0 (PMC10636859; doi:10.1186/s12874-023-02087-0)
Supplement: Supplementary file 2 — Supplementary Material 2 [file 12874_2023_2087_MOESM2_ESM.docx]

**Supplementary file 2：Table 1 Perspectives of different roles on the application of consensus in the guidelines**

| **Perspectives on the consensus process** | **Total （n=312）*** | |  | **Guideline chair(n=71)^&^** | |  | **Working group(n=146)^&^** | |  | **Panel(n=95)^&^** | |
| --- | --- | --- | --- | --- | --- | --- | --- | --- | --- | --- | --- |
|  | **n** | **%** |  | **n** | **%** |  | **n** | **%** |  | **n** | **%** |
| **Methods to form recommendations** |  |  |  |  |  |  |  |  |  |  |  |
| Delphi | 132 | 42.31% |  | 23 | 32.39% |  | 68 | 46.58% |  | 41 | 43.16% |
| Consensus Conference Method | 101 | 32.37% |  | 28 | 39.44% |  | 40 | 27.40% |  | 33 | 34.74% |
| NGT | 39 | 12.50% |  | 3 | 4.23% |  | 22 | 15.07% |  | 14 | 14.74% |
| Modified Delphi | 29 | 9.29% |  | 15 | 21.13% |  | 10 | 6.85% |  | 4 | 4.21% |
| Others | 11 | 3.53% |  | 2 | 2.82% |  | 6 | 4.11% |  | 3 | 3.16% |
| **Understanding of consensus methods** |  | |  |  | |  |  | |  |  | |
| Preliminary understanding | 147 | 47.12% |  | 34 | 47.89% |  | 66 | 45.21% |  | 47 | 49.47% |
| Moderate understanding | 109 | 34.94% |  | 24 | 33.80% |  | 52 | 35.62% |  | 33 | 34.74% |
| Understand very well | 47 | 15.06% |  | 13 | 18.31% |  | 22 | 15.07% |  | 12 | 12.63% |
| Do not know | 9 | 2.88% |  | 0 | 0.00% |  | 6 | 4.11% |  | 3 | 3.16% |
| **Ways to learn consensus methods** |  | |  |  | |  |  | |  |  | |
| Listen to staff reports and meeting reports | 198 | 63.46% |  | 41 | 57.75% |  | 94 | 64.38% |  | 63 | 66.32% |
| Chinese Literature | 196 | 62.82% |  | 47 | 66.20% |  | 90 | 61.64% |  | 59 | 62.11% |
| Participate in relevant training | 172 | 55.13% |  | 50 | 70.42% |  | 85 | 58.22% |  | 37 | 38.95% |
| English literature | 136 | 43.59% |  | 32 | 45.07% |  | 63 | 43.15% |  | 41 | 43.16% |
| others | 13 | 4.17% |  | 2 | 2.82% |  | 6 | 4.11% |  | 5 | 5.26% |
| **How the consensus method is chosen** |  |  |  |  | |  |  | |  |  | |
| Determined by the working group | 81 | 37.33% |  | 20 | 28.17% |  | 61 | 41.78% |  | NA | NA |
| Determined by the chair after the working group proposed | 59 | 27.19% |  | 17 | 23.94% |  | 42 | 28.77% |  | NA | NA |
| Decided after consulting a methodologist | 51 | 23.50% |  | 23 | 32.39% |  | 28 | 19.18% |  | NA | NA |
| Determined by the chair | 24 | 11.06% |  | 11 | 15.49% |  | 13 | 8.90% |  | NA | NA |
| Others | 2 | 0.92% |  | 0 | 0.00% |  | 2 | 1.37% |  | NA | NA |
| **Involvement of methodologists in the consensus process** |  |  |  |  |  |  |  | |  |  | |
| Deep involvement | 197 | 63.14% |  | 32 | 45.07% |  | 100 | 68.49% |  | 65 | 68.42% |
| General involvement | 71 | 22.76% |  | 17 | 23.94% |  | 33 | 22.60% |  | 21 | 22.11% |
| Completely dominated | 25 | 8.01% |  | 7 | 9.86% |  | 11 | 7.53% |  | 7 | 7.37% |
| Partial involvement | 16 | 5.13% |  | 12 | 16.90% |  | 2 | 1.37% |  | 2 | 2.11% |
| Not involved at all | 3 | 0.96% |  | 3 | 4.23% |  | 0 | 0.00% |  | 0 | 0.00% |
| **The importance of face-to-face meetings in the consensus process** |  |  |  |  |  |  |  | |  |  | |
| Necessary | 153 | 49.04% |  | 37 | 52.11% |  | 68 | 46.58% |  | 48 | 50.53% |
| Very necessary | 132 | 42.31% |  | 26 | 36.62% |  | 65 | 44.52% |  | 41 | 43.16% |
| Very unnecessary | 13 | 4.17% |  | 6 | 8.45% |  | 4 | 2.74% |  | 3 | 3.16% |
| Be indifferent | 11 | 3.53% |  | 1 | 1.41% |  | 7 | 4.79% |  | 3 | 3.16% |
| Unnecessary | 3 | 0.96% |  | 1 | 1.41% |  | 2 | 1.37% |  | 0 | 0.00% |
| **Who should lead the consensus meeting process** |  |  |  |  |  |  |  | |  |  | |
| Guideline chair | 161 | 51.60% |  | 46 | 64.79% |  | 70 | 47.95% |  | 45 | 47.37% |
| Working group | 96 | 30.77% |  | 14 | 19.72% |  | 47 | 32.19% |  | 35 | 36.84% |
| Methodologist | 52 | 16.67% |  | 11 | 15.49% |  | 26 | 17.81% |  | 15 | 15.79% |
| Others | 3 | 0.96% |  | 0 | 0.00% |  | 3 | 2.05% |  | 0 | 0.00% |
| **Do you agree with the panel to establish multiple chairs** |  |  |  |  |  |  |  | |  |  | |
| Agree | 209 | 66.99% |  | 49 | 69.01% |  | 94 | 64.38% |  | 66 | 69.47% |
| Strongly agree | 51 | 16.35% |  | 6 | 8.45% |  | 30 | 20.55% |  | 15 | 15.79% |
| Be indifferent | 26 | 8.33% |  | 5 | 7.04% |  | 13 | 8.90% |  | 8 | 8.42% |
| Disagree | 20 | 6.41% |  | 10 | 14.08% |  | 7 | 4.79% |  | 3 | 3.16% |
| Strongly disagree | 6 | 1.92% |  | 1 | 1.41% |  | 2 | 1.37% |  | 3 | 3.16% |
| **Do you agree to conduct a survey on the knowledge background and willingness to participate in the panel?** |  |  |  |  |  |  |  | |  |  | |
| Agree | 202 | 64.74% |  | 46 | 64.79% |  | 91 | 62.33% |  | 65 | 68.42% |
| Strongly agree | 93 | 29.81% |  | 20 | 28.17% |  | 49 | 33.56% |  | 24 | 25.26% |
| Be indifferent | 9 | 2.88% |  | 3 | 4.23% |  | 4 | 2.74% |  | 2 | 2.11% |
| Strongly disagree | 5 | 1.60% |  | 1 | 1.41% |  | 1 | 0.68% |  | 3 | 3.16% |
| Disagree | 3 | 0.96% |  | 1 | 1.41% |  | 1 | 0.68% |  | 1 | 1.05% |

*: Since we permitted participants to complete different roles that applied to them, a total of 312 responses were completed by 290 individuals. In cases where responses from other roles are missing, the calculation will be based on the actual total number of participants.

&: As the topics set by the external reviewer differ significantly from those of the other three roles, this table does not include them.

NA: The questionnaire does not contain any corresponding questions.
