# Supplementary material for: How well did the consensus methods apply in the guideline development of traditional Chinese medicine: a web-based survey in China
Source: BMC Med Res Methodol. 2023 Nov 10;23:264. doi: 10.1186/s12874-023-02087-0 (PMC10636859; doi:10.1186/s12874-023-02087-0)
Supplement: Supplementary file 3 — Supplementary Material 3 [file 12874_2023_2087_MOESM3_ESM.docx]

**Supplementary file 3 Table 2 The most common situations encountered by the guideline chairs during the consensus process**

| **Have you encountered the following situations during the consensus process?(for chairs)** | **n** | **%** |
| --- | --- | --- |
| Late response to the questionnaire | 52 | 73.24% |
| Experts are not good at listening | 43 | 60.56% |
| Ineffective communication due to different knowledge backgrounds | 41 | 57.75% |
| Multiple rounds of discussions fail to reach a consensus | 26 | 36.62% |
| Contradictory opinions of different roles | 25 | 35.21% |
| Experts withdraw from the consensus panel | 2 | 2.82% |
