# Supplementary material for: How well did the consensus methods apply in the guideline development of traditional Chinese medicine: a web-based survey in China
Source: BMC Med Res Methodol. 2023 Nov 10;23:264. doi: 10.1186/s12874-023-02087-0 (PMC10636859; doi:10.1186/s12874-023-02087-0)
Supplement: Supplementary file 4 — Supplementary Material 4 [file 12874_2023_2087_MOESM4_ESM.docx]

**Supplementary file 4 Table 3 Challenges encountered by the panels during the consensus process**

| **Challenges encountered in the process of consensus (for panels)** | **n** | **%** |
| --- | --- | --- |
| Experts are not good at listening to each other | 43 | 45.26% |
| The materials sorted out by the working group are incomprehensible | 40 | 42.11% |
| Ineffective communication due to different knowledge backgrounds | 40 | 42.11% |
| Contradictory opinions of different roles | 37 | 38.95% |
| The evidence gathered by the working group was insufficient | 33 | 34.74% |
| Failed to reply to the questionnaire in time | 24 | 25.26% |
| Multiple rounds of discussions fail to reach a consensus | 23 | 24.21% |
| Views were not heard | 18 | 18.95% |
| No anonymity, dare not comment | 15 | 15.79% |
| The obvious errors in the recommendation were not corrected | 11 | 11.58% |
| Can not withdraw from the Panel | 9 | 9.47% |
| Dissatisfied with the consensus process and quit | 3 | 3.16% |
