# Supplementary material for: How well did the consensus methods apply in the guideline development of traditional Chinese medicine: a web-based survey in China
Source: BMC Med Res Methodol. 2023 Nov 10;23:264. doi: 10.1186/s12874-023-02087-0 (PMC10636859; doi:10.1186/s12874-023-02087-0)
Supplement: Supplementary file 5 — Supplementary Material 5 [file 12874_2023_2087_MOESM5_ESM.docx]

**Supplementary file 5 Table 4 Challenges encountered by the working groups during the consensus process**

| **Challenges encountered in the process of consensus (for working groups)** | **n** | **%** |
| --- | --- | --- |
| Contradictory opinions of different roles | 85 | 58.22% |
| Ineffective communication due to different knowledge backgrounds | 76 | 52.05% |
| Reply to the questionnaire was not timely | 70 | 47.95% |
| Insufficient preparation of consensus related materials | 67 | 45.89% |
| Experts do not understand the concept of PICO* | 62 | 42.47% |
| Experts are not good at listening | 55 | 37.67% |
| Multiple rounds of discussions fail to reach a consensus | 46 | 31.51% |
| The expert's reply was vague | 37 | 25.34% |
| Consensus process record is not detailed | 32 | 21.92% |
| The cooperation of the working group is not tacit | 29 | 19.86% |
| Improper data statistics | 28 | 19.18% |
| Jet lag led to the absence of experts | 16 | 10.96% |
| Experts withdraw from the consensus panel | 9 | 6.16% |

*:Population,Intervention,Comparison and Outcome
